# PASS-predicted Vitex negundo activity: antioxidant and antiproliferative properties on human hepatoma cells-an in vitro study

**DOI:** 10.1186/1472-6882-13-343

**Published:** 2013-12-04

**Authors:** Farkaad A Kadir, Normadiah M Kassim, Mahmood A Abdulla, Wageeh A Yehye

**Affiliations:** 1 Department of Anatomy, Faculty of Medicine, University of Malaya, 50603, Kuala Lumpur, Malaysia; 2 Department of Biomedical Science, Faculty of Medicine, University of Malaya, 50603, Kuala Lumpur, Malaysia; 3 Nanotechnology & Catalysis Research Centre, (NANOCAT), University of Malaya, Block 3A, Institute of Postgraduate Studies Building, 50603, Kuala Lumpur, Malaysia

**Keywords:** Antioxidant, Caspase 3, HepG2, LDH, MTT, PASS, *Vitex negundo*, WRL68

## Abstract

**Background:**

Hepatocellular carcinoma is a common type of tumour worldwide with a high mortality rate and with low response to current cytotoxic and chemotherapeutic drugs. The prediction of activity spectra for the substances (PASS) software, which predicted that more than 300 pharmacological effects, biological and biochemical mechanisms based on the structural formula of the substance was efficiently used in this study to reveal new multitalented actions for *Vitex negundo* (VN) constituents.

**Methods:**

Experimental studies based on antioxidant and antiproliferative assays verified the predictions obtained by the PASS-predicted design strategy. Antioxidant activity of VN extract was studied using 1,1-diphenyl-2-picrylhydrazyl (DPPH) and Ferric reducing or antioxidant power (FRAP) assays. The antiproliferative activity of VN extract against WRL68 and HepG2 was investigated based on methylthiazol tetrazolium (MTT) spectrophotometric assay.

**Results:**

VN extract showed 79.43% inhibition of DPPH stable radical with IC_50_ 13.31 ± 0.18 μg/ml. This inhibition was too closed to butylated hydroxyl toluene (BHT) 82.53% (IC_50_13.8 ± 0.14) and gallic acid 89.51% (IC_50_ 3.1 ± 0.08). VN extract exhibited the strongest free radical scavenging power compared with two commercial antioxidants, BHT and ascorbic acid. VN increased the activities of antioxidant enzymes in normal embryonic liver cells (WRL68) including, superoxide dismutase (SOD) and glutathione peroxidase (GPX) compared with to H_2_O_2_ group. The ethanolic extract of VN showed cytotoxicity to HepG2 cells in a dose and time-dependent manner with IC_50_ 66.46 μg/ml, 57.36 μg/ml and 65.12 μg/ml at 24, 48, and 72-hours incubation respectively, with no sensitivity in WRL68 cells. This was associated with significant elevation in lactate dehydrogenase (LDH) release in HepG2 cells. In addition, the activation of caspase-3 enzyme suggesting that the observed cytotoxicity was mediated via an intrinsic apoptosis pathway.

**Conclusions:**

PASS-predicted plant activity could efficiently help in selecting a promising pharmaceutical leads with high accuracy and required antioxidant and antiproliferative properties. This is the first report on PASS-predicted VN activity.

## Background

Hepatocellular carcinoma (HCC) is a frequent tumour worldwide. It approximately accounts for 6% of cancer occurrences among human and overall, it rates as the seventh most common malignancy in males and the ninth most in females [1]. At least, one million new cases of HCC occur annually and mortality of the disease remains high despite the treatment especially in Southeast Asia countries and tropical Africa, which show the highest incidence [2]. Substantial advances have been applied in the chemotherapy regimen for treating patients with HCC; however, still there is an urge to discover and explore effective strategies for its treatment throughout the use of medicinal plants. Some of the most effective cancer treatments to date are natural products or compounds derived from natural products [3]. Therefore, it is common that there was increasing to find the biological activity among plants with approved medicinal uses rather than from plants randomly selected.

Due to absence of an effective chemotherapy for liver cancer, many studies using different cell lines, animal models and human epidemiological trials have been shown to have considerable potential of herbal medicine to act as anti-proliferative agents and have received a special attention lately [4]. To date, there have been several large (at least 7000 participants) trials testing the efficacy of antioxidant supplements in preventing cancer. A recent review of available literature suggests antioxidants function to prevent free radical damage, and that’s important in preventing our bodies from cancers, arthritis, diabetes, autoimmune diseases and 90+ other diseases [5]. The antioxidant activity of phenolics is mainly due to their redox properties, which allow them to act as reducing agents, hydrogen donors, singlet oxygen quenchers and metal chelators. Meanwhile, the strategy for research and *in vitro* evaluation of biological activity of natural products has changed in the past few years.

VN is a perfect example of medicinal plant credited by numerous medicinal qualities validated by modern science and used since ancient times. It belongs to the family of *Verbenceae,* and the genus consists of 250 species and most of them have commercial and medicinal importance [6]. It is a large aromatic shrub and commonly called five leaved chaste tree or Monk’s Pepper [7]. The leaves contain an alkaloid, flavonoids like flavones, luteolin-7-glucoside, casticin, iridoid, glycosides, an essential oil and other constituents like vitamin C, carotene, glucononital, benzoic acid, β-sitosterol and glycoside [6]. The plant has a pungent, bitter, acrid taste used in various traditional treatment of stomach-ache, disease of the eye, inflammation, enlargement of spleen, bronchitis, asthma, as an antihelmintic, to promote growth of hair and painful teething in children [8].

The isolated compound from the VN extract showed protection of hepatocytes, nephrocytes and pancreatic β-cells probably by its action against NF-kB and induced- nitric oxide synthase iNOS mediated inflammation in streptozotocin-induced diabetes [9]. VN seeds extract showed analgesic, antinociceptive activity [10] and hepatoprotective activity [11-13].

Apoptosis is a type of programmed cell death; it allows proper development and remodeling of normal tissues, besides generating immune responses and destroying abnormal cells. It is well known that malignant transformation of human cancer cells is a multi-staged process involving mutation or deletions of various human suppressor’s genes. This can lead to activation of oncogenes and alterations in the level of expression of key regulatory genes, providing growth advantages and metastatic potential for tumor cells [14]. Those genetic alterations result in abnormalities in cellular and nuclear morphology and signal transduction pathways. This would normally activate a suicidal pathway and induce apoptosis in the cells leading to defects and/or mutations of p53 delay cell-cycle arrest and abolish the DNA repair process, which otherwise render the cells to apoptosis [2]. Virtual screening is of specific significance to understand the pharmacological action of the plant compounds [15]. The prediction of activity spectra for substances (PASS) software [16], predicted more than 300 pharmacological effects, biological and biochemical mechanisms based on the structural formula of the substance. This was efficiently used in this study to reveal new multitalented actions for the isolated components of VN extract.

HepG2 cell line represents one of the most widely used experimental model for *in vitro* studies on HCC [17]. Hence, this work was carried out to investigate the antioxidant, antiproliferative and apoptotic effect of ethanolic extract of VN extract against WRL68 and HepG2 cell lines based primarily on the rich literature review with the support of PASS prediction program.

## Methods

### Computational evaluation of biological activity

The biological activity spectra of the phytoconstituents for VN extract were obtained using the Prediction of Activity Spectra for Substances (PASS) software. PASS prediction tool is constructed using 20,000 principal compounds from the MDDR database (produced by Accelrys and Prous Science) [18].

### Preparation of crude ethanol extract

Fresh leaves of VN plant were obtained from Kampung Baru, Sungai Ara, Penang, Malaysia. The plant was identified and the voucher specimen number (KLU 34968) was deposited in University Malaya (Department of Pharmacy). Dried and ground leaves of VN were weighed, then homogenized in 95% ethanol at a ratio of 1:10 of plant to ethanol and left to soak for 4 days at 25°C while shaking and stirring it occasionally. The mixture was filtered, centrifuged at 14,000 rpm for 10 min and then concentrated under reduced pressure at 45°C to obtain a dark gummy–green extract. The concentrated extracts were then frozen and finally lyophilized with freeze dryer, yielding the crude extract of the leaves of VN.

### DPPH scavenging assay

The extract was measured in terms of hydrogen donating or radical scavenging ability using the stable radical DPPH following the method described by Gorinstein et al., 2003 [19]. The colour change of the reaction mixture was then read at 517 nm against the blank, which did not contain the extract. Galic acid, ascorbic acid and BHT were used as a positive control. Samples without treatment were used as negative control. The percentage of DPPH decolourization of the sample was calculated as

$$\begin{array}{l} {\mathrm{DPPH}}^{\cdot }\mathrm{scavenging}\;\mathrm{effect}\;\left(\%\right)\\ =\left(\mathrm{Control}\;A–\mathrm{Sample}\;A/\mathrm{Control}\;A\right)\times 100 \end{array}$$

Where Control A is the absorbance of the control reaction. Sample A is the absorbance in the presence of VN extract. The test was conducted in triplicate.

### FRAP Assay

The FRAP assay measures the change in absorbance at 593 nm due to the formation of blue coloured Fe^2+^ - tripyridyltriazine (Fe - TPTZ) compound from the colourless oxidized Fe^3+^ form by the action of electron donating antioxidants [20]. The experiment was conducted at 37°C under pH 3.6 condition with a blank sample in parallel. In the FRAP assay, reductants “antioxidants” in the sample reduce Fe (III)/tripyridyltriazine complex, present in stoichiometric excess, to the blue ferrous form, with an increase in absorbance at 593 nm.

Briefly 50 μl from the dissolved extract was added to 1.5 ml freshly prepared and pre warmed (37°C) FRAP reagent (300 mM acetate buffer, pH_3_.6, 10 mM TPTZ in 40 mM HCl and 20 mM FeCl_3_.6H_2_O in the ratio of 10: 1:1) and incubated at 37°C for 10 min. The absorbance of the sample was read against reagent blank (1.5 ml FRAP reagent + 50 μl distilled water) at 593 nm. Increased absorbance of the reaction mixture indicated increased reducing power. Ascorbic acid, galic acid and BHT were used as standards. All analyses were run in triplicate and results averaged.

### *In vitro* VN antioxidant inWRL68 cell lines

The VN extract was used for *in vitro* antioxidant experiment. Approximately, 1000 μl of the WRL-68 cell line suspension were seeded in 12-well flat bottom micro titer plates at 2 × 10^6^ cells/ml in Dulbecco’s Modified Eagle Medium (DMEM ) containing 10% (v/v) FBS and allowed to attach overnight. The second day, the cells were treated with 100 μg of VN extract in triplicate according to Table 1 and incubated at 37°C with 5% CO_2_ for 2 hours. The treated cells were induced by 100 μl of freshly prepared 1000 μM H_2_O_2_ and re-incubated for 2 hours. The H_2_O_2_-treated and untreated cells after removing the medium, were harvested, washed twice with PBS and lysed in lysis buffer (25 mmol/l Tris–HCl). WRL-68 cell lysates were prepared in a 0.5 ml cold phosphate buffer saline (PBS) (pH 7.4) [21]. All the cell debris was removed by centrifugation at 100 rpm for 10 min at 4°C using refrigerated centrifuge Rotofix 32 (Hettich Zentrifugen, Germany). All samples were sonicated for 5 min with 10 sec rest after each min. The samples were kept at −20°C until used. The supernatant was used for the estimation of the following antioxidant using commercially available kits from (Cayman Chemical Company, USA): malondialdehyde (MDA) (Item No. 10009055), superoxide dismutase (Item No. 706002) and glutathione peroxidase (Item No. 703102) activities.

**Table 1:** ***In vitro *** **antioxidant for cell line experimental design**

**Group Name**	**Oxidant agent (1000 μM H**_**2**_**O**_**2**_**)**	**Treatment (100 μg/ml)**
**Normal (untreated cell group)**	No oxidant agent (10 μl medium)	10 μl solvent
**H**_**2**_**O**_**2 **_**(Oxidative damaged group)**	10 μl	10 μl solvent
**VN**	10 μl	VN ethanol extract (10 μl)

### Cell culture

Two types of cells were used; (WRL68) and (HepG2). Both cell types were obtained from Department of Molecular Medicine, Faculty of Medicine, University of Malaya. Cells were cultured in the DMEM, supplemented with 10% fetal bovine serum, penicillin (100 units/ml-streptomycin (100 μg/ml), using 75 cm^2^ flasks in a 37°C in humidified 5% CO_2_ incubator.

### MTT assay

Briefly, the cells were plated into 96-well plates at the density of 1.5 × 10^4^/well in the final volume of 100 μl culture medium per well. On the following day, the cells were treated with various concentration of VN plant extract at doses of 6.25, 12.5, 25, 50,100 and 200 μg/ml and maintained at 37°C with 5% CO_2_ for 24, 48 and 72 hours. Sample without treatment was used as negative control. At the end of the incubation period, 20 μL of MTT reagent was added to each well and incubated again for 4 hours at 37°C with 5% CO_2,_ then 100 μL of dimethylsulphoxide (DMSO) was added into each well and the absorbance was determined at 540 nm using ELISA reader. The cell viability percentage was calculated using the formula:

$$A_{550\mathrm{nm}}\left(\mathrm{sample}\right)/A_{550\mathrm{nm}}\left(\mathrm{control}\right)\;\times \;100$$

Where A (sample) is the absorbance of wells containing different concentrations of plant extract and A (control) is the absorbance of control wells containing cell culture medium without samples. The experiment was carried out in triplicates [22].

### Cell observation using an inverted microscope

HepG2 cell lines were cultured in 96-well plates and treated with VN ethanolic extract*.* The cells were then rinsed with 1× Phosphate Buffer Saline (PBS) (Sigma). Morphological and confluence changes of the cells in VN-treated group 57.36 μg/ml according to IC_50_ and untreated group for 48 hours were observed under × 10 magnification by a trinocular inverted phase contrast microscope (Olympus, Japan).

### Acridine orange/ethidium bromide (AO/EB) staining

Dual staining with acridine orange and ethidium bromide was performed based on the protocol previously described [23]. Cells were seeded in six well plates for 48 hours and subjected to treatment with VN in a dose of 57.36 μg/ml according to IC_50_. After incubation, the cells were harvested by trypsinization and rainsed with PBS, and then stained with 0.1 mg/ml acridine orange and 0.1 mg/ml ethidium bromide. Stained cell suspension (10 μl) was placed on a clean glass slide and covered with a cover slip. The cells were then observed under a fluorescence microscope (Leica) in both red channel (590 nm) and green channel (520-550 nm).

### Lactate dehydrogenase (LDH) assay

To determine the effects of ethanolic extract of VN on membrane permeability in WRL 68 and HepG2 cell lines, LDH release assay was done using (Cayman Chemical Company, USA) LDH Cytotoxicity Assay Kit, (item No. 10008882). The presence of LDH enzyme in the cell culture medium is an indication of cell membrane damage [24]. Basically, LDH cytotoxicity assay kit measures cell death in response to chemical compounds using a coupled two-step reaction. In the first step, LDH catalyzes the reduction of NAD^+^ to NADH and H^+^ by oxidation of lactate to pyruvate. In the second step of the reaction, diphorase uses the newly-formed NADH and H^+^ to catalyze the reduction of a tetrazolium salt (INT) to highly-coloured formazan which absorbs strongly at 490-520 nm. The amount of formazan produced is propotional to the amount of LDH released into the culture medium as a result of cytotoxicity.

The cells were seeded in a 96-well plate at a density of 10^4^-10^5^ cells/well in 120 μl of culture medium with or without compounds to be tested.

### Detection of apoptosis of HepG2 cells by measuring caspase- 3 enzyme activity

Caspase-3 activity was assessed using the caspase-3Colorimetric Assay Kit (BioVison), following the manufacturer’s instructions is based on spectrophotometric detection of the chromophore *p*-nitroaniline (*p*NA) after cleavage of a specific substrate DEVD-pNA.

The HepG2 cells were seeded in sterile 60 mm dishes, and at the end of VN treatment, the cells were washed with PBS and lysed in lysis buffer provided by the kit. After freezing and thawing three times, the cell lysate was centrifuged at 20,000× g at 4°C for 15 minutes. The supernatants were collected and DEVD-pNA was then added and incubated for 1–2 hours at 37°C. The concentration of the pNA released was measured at 405 nm, and the quantity of pNA was calculated from a calibration curve of pNA standard. Caspase-3 activity was expressed spectrophotemetrically compared to the control untreated cells.The experiment was carried out in triplicates [25].

### Statistical analysis

The analysis of variance (ANOVA) was used to determine differences between treated and control groups with *p* < 0.05 being considered as statistically significant, using SPSS programme for Windows version 18.0 (SPSS Inc. Chicago, IL, USA).

## Results and discussion

### PASS prediction and assistant experimental design

In order to accelerate the research for potent natural products, computer-aided drug discovery program PASS was used to predict the antioxidant and antioproliferative properties. PASS prediction tools were constructed using 20000 principal compounds [26] and about 4000 kinds of biological activity on the basis of structural formula with mean accuracy about 90% [27]. The result of prediction is presented as the list of activities with appropriate Pa and Pi ratio.

Pa and Pi are the estimates of probability for the compound to be active and inactive, respectively. It is reasonable that only those types of activities may be revealed by the compound, which Pa > Pi. If Pa > 0.3 the compound is likely to reveal this activity in experiments, but in this case the chance of being the analogue of the known pharmaceutical agents for this compound is also high. Thus, potential biological effects of the plant constituents were predicted by PASS program based on structure activity relationship (SAR) analysis of the training set containing thousands of compounds which have many kinds of biological activity. Therefore, before we started our experiments, we used PASS program to validate whether VN constituents based on SAR strategy is in agreement with the SAR of the training set of the PASS database.

A portion of the predicted biological activity spectra (lipid peroxidase inhibitor, antioxidant, free radical scavenger, hepatoprotectant, caspase-3 stimulant and antiproliferative) for the VN extract isolated compounds are given in Table 2.

**Table 2:** Part of PASS for VN chemical compounds

	**Biological activity**											
**Compound**	**Lipid peroxidation inhibitor**		**Antioxidant**		**Free radical scavengers**		**Hepatoprotectant**		**Caspase-3 stimulant**		**Antiproliferative**	
	**Pa**	**Pi**	**Pa**	**Pi**	**Pa**	**P**	**Pa**	**Pi**	**Pa**	**Pi**	**Pa**	**Pi**
**1**	0.813	0.003	0.699	0.004	0.786	0.003	0.717	0.007	0.741	0.009	0.737	0.012
**2**	0.765	0.004	0.671	0.004	0.765	0.003	0.744	0.006	0.706	0.011	0.754	0.011
**3**	0.798	0.004	0.749	0,004	0.849	0.002	0.741	0.006	0.778	0.007	0.886	0.005
**4**	0.711	0.005	0.644	0.004	0.751	0.003	0.658	0.009	0.734	0.009	0.855	0.005
**5**	0.952	0.002	0.874	0.003	0.973	0.001	0.912	0.002	0.812	0.005	0.817	0.007
**6**	0.304	0.062	0.788	0.003	0.518	0.009	0.983	0.001	0.679	0.012	0.445	0.005
**7**	0.415	0.031	0.598	0.005	0.698	0.004	0.986	0.001	0.611	0.006	0.554	0.014
**8**	0.920	0.002	0.798	0.003	0.956	0.001	0.924	0.002	0.749	0.008	0.715	0.013

Pa—probability “to be active”; Pi—probability “to be inactive”.

**(1)** 5-hydroxy-7,4′dimethoxy flavones, **(2)** 5-hydroxy-3,6,7,3′,4′-Pentamethoxy flavones, **(3)** 5, 3′ dihydroxy-7, 8,4′-trimethoxy flavanone, **(4)** 5,7-dihydroxy-6,4′dimethoxy flavanone, **(5)** 7,8-dimethyl herbacetin 3-rhamnoside, **(6)** Agnuside, **(7)** Negundoside, **(8)** Vitegnoside.

### DPPH assay

**Figure 1:**
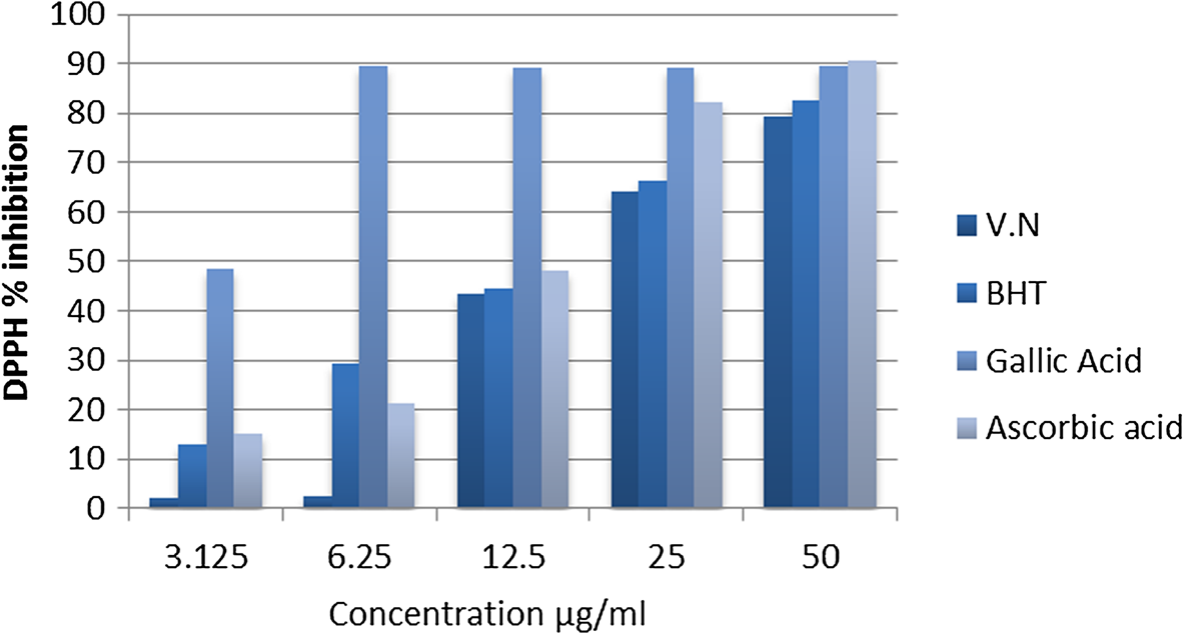
The percentage of scavenging activity of ethanolic extract of VN against DPPH.

The DPPH assay was utilized to evaluate the ability of the investigated VN ethanolic extract to act as donors of hydrogen atoms or electrons in transformation of DPPH radical into its reduced form DPPH-H. The extract of VN was able to reduce the stable purple-coloured radical DPPH into the yellow-colored DPPH-H. The scavenging activities of VN extract, ascorbic acid, galic acid and BHT on DPPH radicals were compared as shown in Figure 1. Percentage of radical scavenging activity at the highest concentration was 79.43 ± 1.3 for VN, BHT 82.53 ± 1.7, galic acid 89.51 ± 1.14 and ascorbic acid 90.65 ± 1.34.

### Ferric Reducing antioxidant power (FRAP) of VN extract

**Figure 2:**
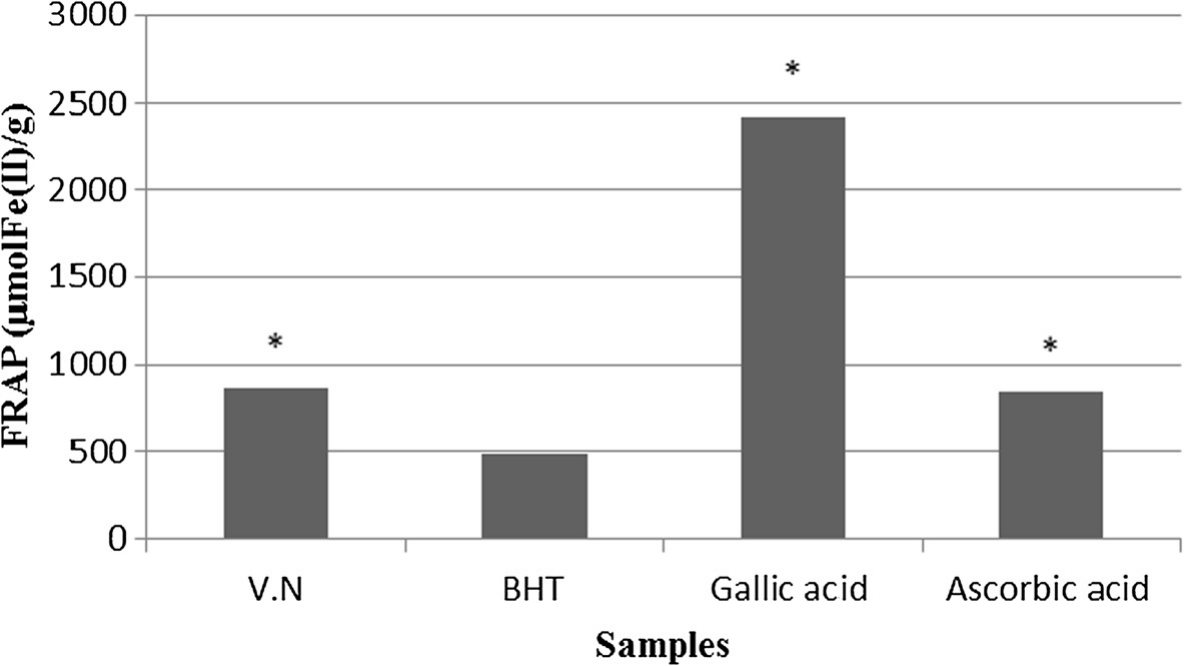
**Ferric reducing antioxidant property (FRAP) of the leave extract of VN.** Values are presented as means ± SEM, * P < 0.05 versus BHT.

The reducing ability of the VN extract was in the range of 866.11 μmol Fe (II)/g. The FRAP value for VN extract was lower than ascorbic acid and galic acid, but significantly higher than BHT (Figure 2).

The phenolic components most frequently represented in ethanol extracts from VN: negundoside, agnuside, vitegnoside, 7,8 dimethyl herbacetin 3-rhamnoside, 5,3′-dihydroxy—7,8,4′-trimethoxy flavanone, 5-hydroxy-3,6,7,3′,4′-pentamethoxy flavone, 5,7 dihydroxy- 6,4′ dimethoxy flavonone, and 5 hydroxy-7,4′ dimethoxy flavone, and among these, negundoside is the most active phenol acting as an antioxidant. It can protect against CCl_4_-induced toxicity and oxidative stress. The mechanism of protection involves decreased production of ROS and lipid peroxidation. The agnuside, vitegnoside and flavonoids present in the plants are also natural antioxidants [12,13].

**Figure 3:**
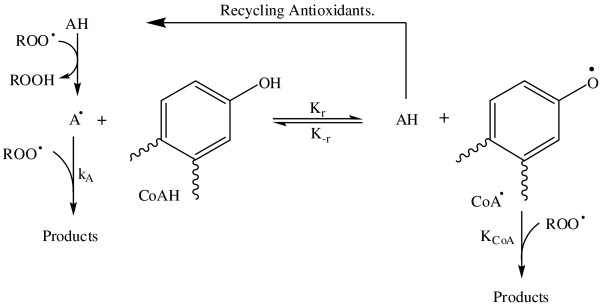
**Co-antioxidant effectively recycles antioxidant.** AH = antioxidants, α-TOH; CoAH = co-antioxidant.

VN extract showed significant levels of % inhibition of DPPH˙(79.43 ±1.3) compared to the standard antioxidants (BHT, gallic acid and ascorbic acid) which were used as positive control (Table 3). As shown in this table, VN extract was significantly (p < 0.05) lower than BHT, galic acid and ascorbic acid at low concentrations, 3.125 and 6.25 μg/ml giving lowest inhibition at 2.35% and 2.58%, respectively. Interestingly, this inhibition was significantly increased by the increasing the concentration from 12.3 to 25 to 50 μg/ml to give 79.43% with IC_50_ 13.31 ± 0.18 μg/L (Table 3). This inhibition is too closed to BHT 82.53% with IC_50_ 13.8 ± 0.14 μg/L and gallic acid 89.51% with IC_50_ 3.1 ± 0.08 μg/L. This clearly indicates that the VN extract has good radical scavenging activity compared to the pure compounds. Presumably, significantly greater inhibition of DPPH could be attributed to the presence of multi-hydroxyl groups, which is the active center of anti-oxidation like7, 8 dimethyl herbacetin 3-rhamnoside and vitegnoside which showed radical scavenging activity 97.3% and 95.6% respectively (Table 2). Although we do not have an exact explanation for the greater increase of VN free radical scavenging activity, we could provide some logical arguments. Our published data [13] showed that VN is rich in phenolic compounds and provides a wide range of antioxidant properties which seems to be directly related to the hydroxyl groups attached to aromatic rings. This broad spectrum of antioxidant formula provides the best possible protection against the free radicals. This attributed to bond dissociation energy (BDE) of each hydroxyl group attached to the benzene ring. It is the energy needed to break one mole of the bond to give separated atoms. BDE plays a central role in determining antioxidant efficacy, and is one of the most important physical parameters used for evaluating antioxidant activity in chemical compounds that are used as inhibitors of free radical reactions [28]. In general, compounds having lower BDEs have been reported to have better antioxidant properties. Hence, VN has a wide variety of hydroxyl groups and therefore, exerts a wide variety of BDEs, forming a synergistic system between antioxidant and co-antioxidant by regeneration of antioxidants through the co-antioxidant. For instance, vitamin E (α-TOH) and polyphenols can be involved in the synergistic antioxidant system. α-TOH is consumed from the beginning of the oxidation reaction forming α-TO**˙** radical which completely preserved until all co-antioxidant (CoAH) has been consumed (Figure 3). Due to this peculiar behavior, polyphenolic species are ideal co-antioxidants to be used together with a small amount of vitamin E [29,30].

**Table 3:** **IC**_**50 **_**value and percentage inhibition of the DPPH radical scavenging assay**

**μg/ml**	**Max. inhibition% ± SEM **^**a**^			
	**VN**	**BHT**	**Gallic acid**	**Ascorbic acid**
**3.125**	2.35 ±0.07	13.05 ±0.07	48.4 ± 0.01	15.1 ±0.14
**6.25**	2.58 ± 0.4	29.15 ±0.21	89.3 ±0.51	21.24 ±0.86
**12.3**	43.5 0.47	44.5 ±0.07	89.1 ±1.27	48.06 ±1.18
**25**	64.2 ±0.94	66.32 ±0.96	89.17 ±1.65	82.09 ±1.09
**50**	79.43 ±1.3	82.53 ±1.7	89.51 ±1.14	90.65 ±1.34
**IC50 **^**b **^**value μg/l ± S.E.M**	13.31 ± 0.18	13.8 ± 0.14	3.1 ± 0.08	12.9 ± 0.12

^a^SEM: standard error of the mean. ^b^IC_50_: 50% effective concentration. IC_50_ is determined by using Masterplex 2010 software (5Parameters logistics).

The FRAP assay measures the ferric [Fe^3+^-TPTZ]-to-ferrous [Fe^2+^-TPTZ] iron reduction in the presence of antioxidants. FRAP assay treats the antioxidants in the sample as a reductant in a redox-linked colorimetric reaction [31]. The trend for ferric ion reducing activity of VN against BHT, gallic acid and ascorbic acid are shown in Figure 2. VN exhibited the strongest free radical scavenging power compared with two commercial antioxidants, BHT and ascorbic acid. This seems to suggest that VN extract can donate electron easily. This activity is believed to be mainly due to their redox properties. Hence VN extract should be able to donate electrons to free radicals stable in the actual biological and food system.

The ethanolic extract of VN was found to be an effective scavenger of DPPH and FRAP with a good reducing power activity. The high antioxidant activity of VN enhanced the potential interest in this plant for improving the efficacy of different products as nutraceutical and pharmacological agents.

### *In vitro* antioxidant of VN for WRL68 cell lines

The oxidative stress was induced by exposing cells to 1000 μM H_2_O_2_ for 2 hours while the protective effect of the plant reduce the oxidative stress. Cells were first pre-incubated with VN for 2 hours and then treated with 1000 μM H_2_O_2_. It is obvious that H_2_O_2_ lead to the production of reactive oxygen species (ROS), which in consequence reduced the antioxidant enzymes such as SOD and GPX. However, pre-treatment with plant extract decreased the free radical formation; therefore the antioxidant enzymes level became higher. Our results revealed that H_2_O_2_-exposed cells caused a statistically significant decrease (p < 0.05) in GPX activity, whereas those exposed to VN showed significant increase in GPX activity. On the other hand, lipid peroxidation value, measured as MDA production, was significantly increased in H_2_O_2_- induced oxidative stress group compared to untreated cells. Whereas in cells pre-incubated with VN extract, there was significant reduction in MDA level due to prevention of lipid peroxidation (Table 4). This is probably due to the presence of 7, 8 dimethyl herbacetin 3-rhamnoside and vitegnoside which showed the highest lipid peroxidase inhibitor activity in PASS result (Table 2).

**Table 4:** **Effects of H**_**2**_**O**_**2**_**, VN extract on the antioxidant enzymes and MDA level on H**_**2**_**O**_**2 **_**– induced WRL-68 cell line**

**Group**	**SOD (U/mg protein )**	**GPX (nmol/ mg protein )**	**MDA (nmol/ g protein )**
**Normal Control**	10.61 ± 0.010	27.14 ± 0.30	17.00 ± 1.019
**H**_**2**_**O**_**2**_	10.00 ± 0.065	10.98 ± 0.18^a^	46.94 ± 1.730^a^
**VN extract**	10.37 ± 0.150	22.05 ± 0.19^b^	23.17 ± 2.165^b^

Values are presented as means ± S.E.M, ^a^*P* < 0.05 versus normal control group, ^b^*P* < 0.05 versus H_2_O_2_ group.

The role of oxidative stress and tissue damage in diseases, such as atherosclerosis, heart failure, neurodegenerative disorders, aging diabetes mellitus, hypertension and other several diseases are gaining a lot of recognition [32]. Reactive oxygen species (ROS) are prospective carcinogenic substances because of the generating free radicals including hydroxyl, superoxide, peroxyl, hydroperoxyl, and alkoxyl radicals, which participate in tumor promotion, mutagenesis and progression. If there is no effective regulation, the excess ROS will damage proteins, lipids or DNA and in turn inhibition of the normal function through the modulation of gene expression, cell cycle, cell metabolism, cell adhesion and cell death [33].

Glutathione is the major endogenous antioxidant scavenger that protects cells from oxidative stress through its ability to bind to and reduce ROS [34]. Thus, preserving the glutathione-mediated antioxidant defense is critical for cell survival. Glutathione is formed by γ-glutamate cysteine ligase (γ -GCL) and glutathione synthetase [35]. The ethanol extract of VN increased the GPX and SOD activity, which indicates that this extract can effectively scavenge H_2_O_2_. The effects of the ethanol extract of VN on cell viability might involve dual actions: the direct action of oxygen radical scavenging, as shown by the DPPH radical scavenging by ethanol extract (Figure 1) and the indirect action via the induction of the antioxidant enzymes of SOD and GPX (Table 4). Furthermore, the level of lipid peroxidation was significantly higher in the cells exposed to H_2_O_2_, while the treatment with VN extract apparently attenuated the MDA level. This might reflect an idiosyncrasy of the *in vitro* system used in this study.

### Cytotoxic effect of VN extract on HepG2 and WRL 68 cell lines

**Figure 4:**
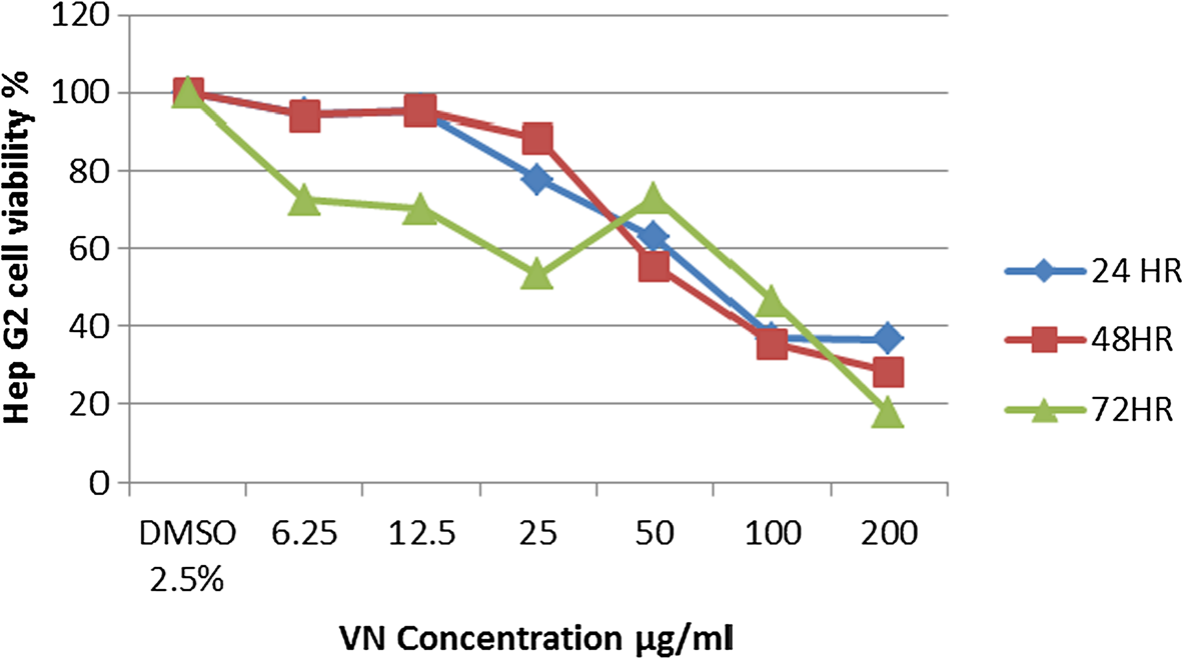
Effect of VN treatment on HepG2 cell lines viability.

Cytotoxicity of VN crud extract on HepG2 and WRL68 cells was assessed using MTT assay. Responses of HepG2 cells toward increasing concentrations of VN extract were exponential. HepG2 cells experienced a significant increase in inhibition at low concentrations of VN extract, with an eventual decline at the highest concentrations tested and with the increasing in the incubation period. The estimated IC_50_ values of VN extract were 66.46 μg/ml, 57.36 μg/ml and 65.12 μg/ml at 24 hours, 48 hours and 72 hours incubation respectively (Table 5). This means that increasing the concentration used combined with a longer period of incubation with VN extract has an impact on increasing the ability of inhibition of proliferation. This is indicated by the declining number of living cells with increasing concentrations and incubation time of HepG2 cells (Figure 4).

**Table 5:** **Comparison of IC**_**50 **_**values for HepG2 and WRL 68 cells obtained from MTT assay following exposure to VN extract for 24,48 and 72 hours**

**Cell line**	**Extract**	**Duration(Hour)**	**IC**_**50 **_**μg/ml**
WRL 68	VN	24	>100 ^a^
		48	>100 ^a^
		72	>100 ^a^
Hep G2	VN	24	66.46 ± 2.8
		48	57.36 ± 1.3
		72	65.12 ± 1.8

The IC_50_ value is defined as the concentration of sample necessary to inhibit 50% of the cells. ^a^Value not reach 50% inhibition. IC_50_ is determined by using Masterplex 2010 software (5Parameters logistics).

**Figure 5:**
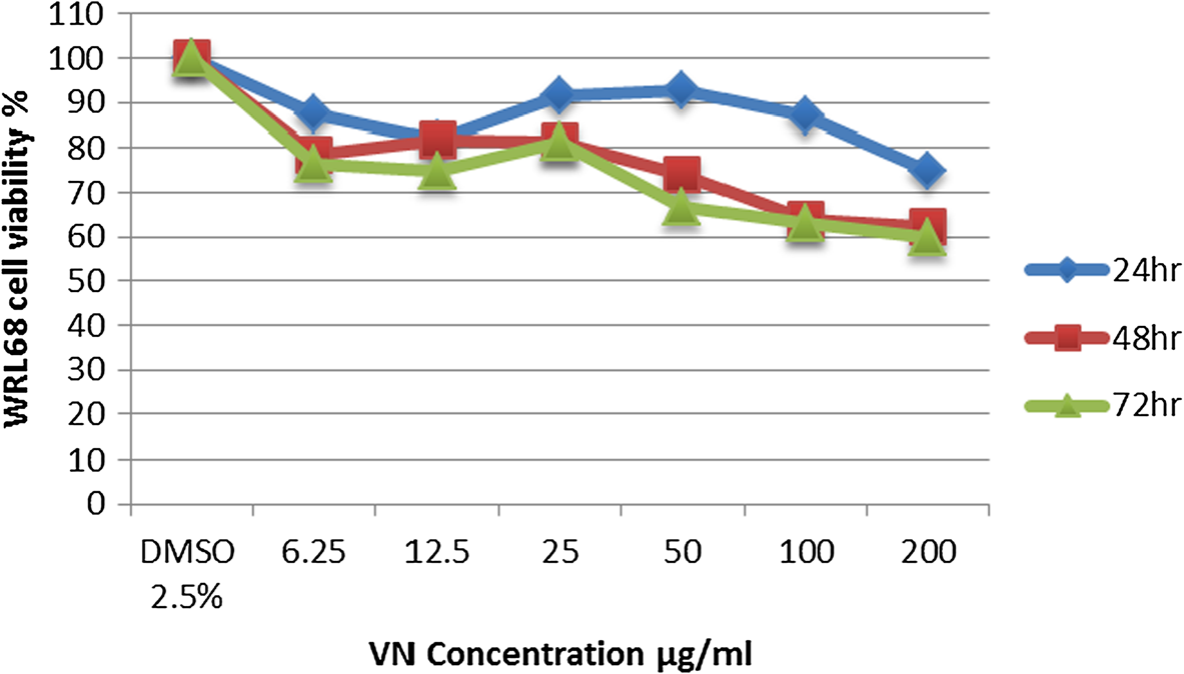
Effect of VN treatment on WRL68 cell lines viability.

The cytotoxicity or anticancer activity of the crude extract expressed as the inhibitory of concentration (IC_50_). The sensitivity of HepG2 cells to VN is characterized by IC_50._ The lower the IC_50_ value indicated the higher anticancer effect of the sample. These results indicate that elevated anticancer effects strengthened with dose time of exposure (Table 5).

(Figure 5) showed that (WRL68) cells treated with 200 μg/ml of VN ethanolic extract still retained > 50% viable cells 59.86% viability. Hence, VN ethanolic extract predetermination by MTT assay induced cytotoxicity activity in the (HepG2), but not in (WRL68) cells.

Until now, no ideal cytotoxicity test has been developed; hence, in this study, we have screened this type of plant which is native to South Eastern Asian countries for treating a variety of ailments, including cancer by using two-cell lines, WRL68 and HepG2 cells. The micro-culture tetrazolium salt (MTT) assay was used in this study to measure the amount of cell viability. This method is based on the quantification of purple-coloured formazan, which was formed by the reduction of MTT [3-(4, 5-dimethylthiazole-2-yl)-2,5-diphenyl. The potential antiproliferative effect of ethanolic crude extract of VN was investigated, determining their effect on the viability of (HepG2) and (WRL 68). The reduction of MTT is proportional to the number of active mitochondria in the live cells. The results indicated that VN extract caused significant inhibition of HepG2 cells in a dose and time-dependent manner. Generally, it was found that VN extract at IC_50_ 57.36 μg/ml was cytotoxic for HepG2 cell lines after 48 hours of exposure, meanwhile, there was no IC_50_ value of VN extract against WRL 68 cell lines in all concentrations and incubation periods as shown in Table 5.

### Morphological observation by inverted microscope

**Figure 6:**
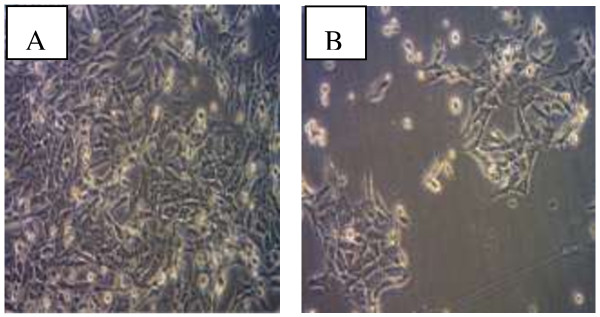
**Photomicrographs showing the comparison of the morphology of HepG2 cells.** Before and after treatment with VN extract **(A)** untreated cells (normal control). **(B)** VN treated cells, (magnification × 10).

Observation through a light microscope of the VN ethanolic extract-treated HepG2 cell line after 48 hours of exposure showed typical morphological features of apoptosis. The characterization of morphological changes observed showed reduction in cell volume, cell shrinkage, reduction in chromatin condensation and formation of cytoplasmic blebs as shown in Figure 6.

### Morphological observation by acridine orange and ethidium bromide (AO/EB) staining

**Figure 7:**
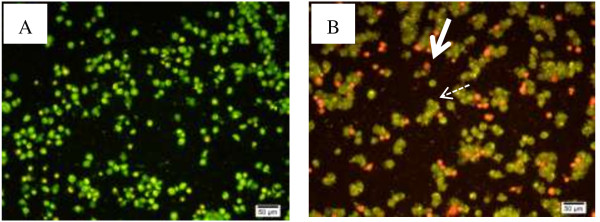
**Effect of VN and CS on morphology of HepG2 cells. (A)** Untreated controlled cells. **(B) **VN treated cells, full white head arrows: apoptotic cells and dashed dotted arrow indicated cell with fragmented nuclei. AO/EB staining, fluorescence microscope, Bar = 50 μm.

Induction of apoptotic cell death among HepG2 cells was confirmed by a morphological observation of the cells after Acridine Orange/Ethidium Bromide (AO/EB) staining assessed by fluorescence microscopy. As shown in Figure 7, the cells treated with VN showed a marked decrease in the number of live cells compared to the untreated control.

### LDH Release

**Figure 8:**
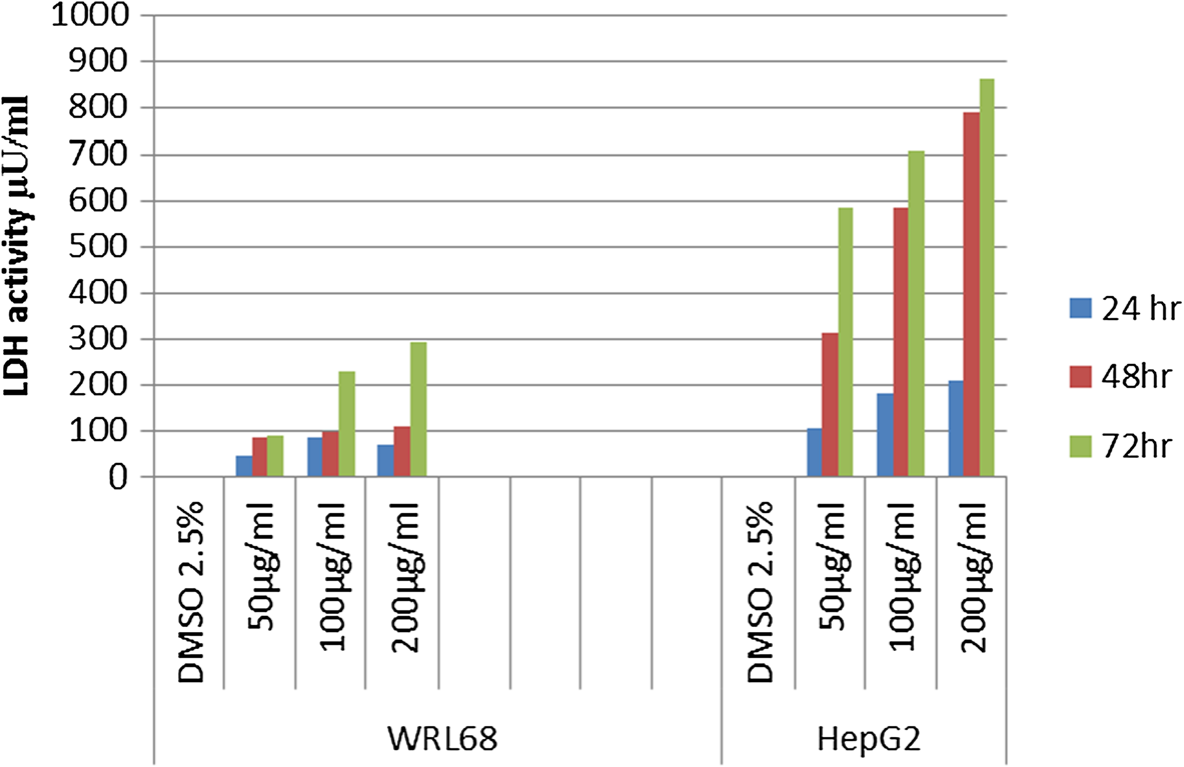
Effect of VN treatment in WRL68 and HepG2 cell lines on LDH release.

LDH is an enzyme located in the cell and catalyses the interconversion of lactate to pyruvate. It is another indicator of cell viability through the evaluation of membrane integrity. The LDH activity is measured externally as it leaks from dead cells or disturbed cells and in both cases the LDH activity increases. In the present study, LDH assay was carried out to evaluate the *in vitro* cytotoxic effect of VN extract on WRL 68 and HepG2 cells. (Figure 8) showed that the lowest LDH release was in WRL 68 cells at 50 μg/ml within 24 hours incubation period and it was activated with the increase of VN concentration and the incubation period.

On the other hand, HepG2 cells were more sensitive to VN as compared to WRL 68 cells, LDH was released in a time and dose dependent manner.

Recent studies have shown that LDH is an accurate and more reliable marker of cytotoxicity, because damaged cells are fragmented completely during the higher concentration of VN extract and the path of prolonged incubation with substances [36,37]. In this report, LDH amount from HepG2 cells was released in a time and dose dependent manner as shown in Figure 8 and this indicted that VN extract is less cytotoxic on WRL 68 cell lines and the highest concentration of VN (200 μg/ml) showed the highest toxicity on both cell lines. Based on MTT spectrophotometric assay, VN showed high antiproliferative activities toward HepG2 cell lines in a dose and time-dependent manner Figure 4A. The sensitivity of HepG2 cells to VN is characterized by IC_50_ value. These results indicate that elevated antiproliferative effects strengthened with the dose and time of exposure. This was based on the average of three sets of experiments. To prove that the apoptosis has been influenced by VN ethanolic extract, HepG2 cells were examined in the presence of acridine orange and ethidium bromide staining (AO/EB staining). Acridine orange is a vital dye that will stain both live and dead cells, whereas ethidium bromide will stain only those cells that have lost their membrane integrity [38]. Cells stained green stand for viable cells, whereas reddish or orange staining illustrates late apoptotic cells. As shown in Figure 7, HepG2 cells treated with 200 μg/ml of ethanolic extract showed changes in cellular morphology, including chromatin condensation, membrane blebbing, and fragmented nuclei. Therefore, we can assume that stronger apoptosis is associated with high concentration of VN extract.

### Caspase-3 activity

**Figure 9:**
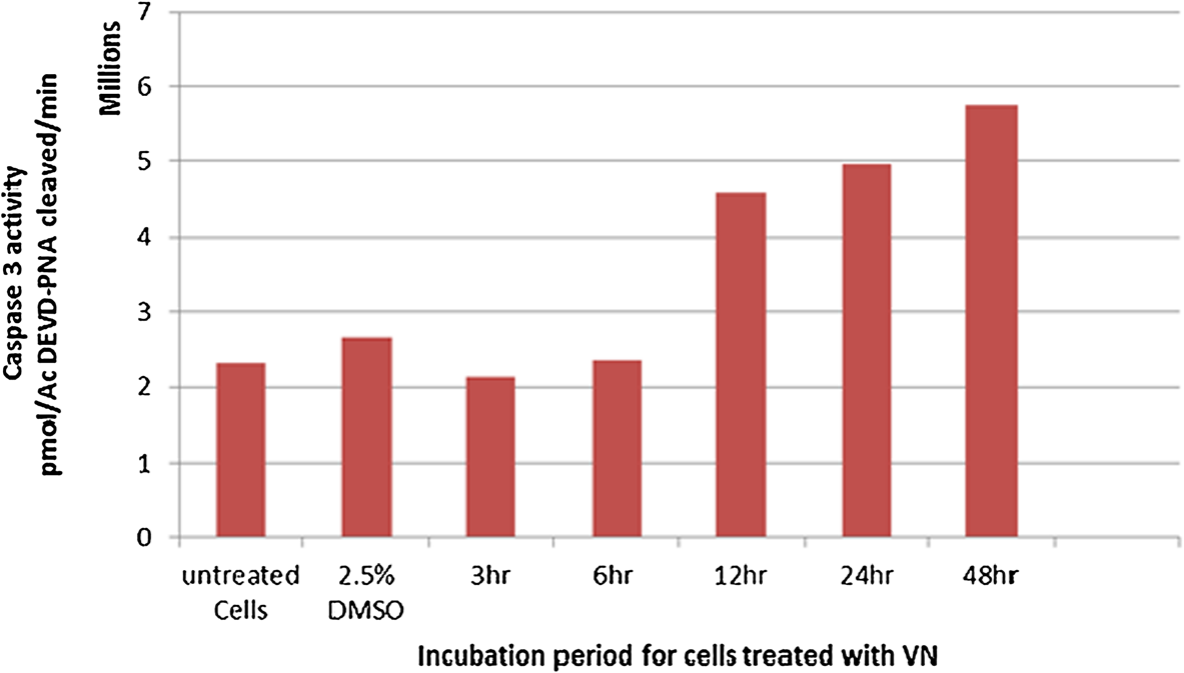
Caspase-3 activity of HepG2 cells treated with VN.

Caspase-3 activation is a vital element in the apoptotic signaling cascade. Although VN was not choicely cytotoxic to HepG2 cells, we were enthusiastic over check if the cytotoxicity to HepG2 cells treated with VN was mediated by apoptosis. To further elucidate the mechanism of cell death induced by VN, a caspase-3 colorimetric assay was conducted to establish the levels of caspase-3 activation both before and after treatment with the extract. The results of this experiment showed that treatment of HepG2 cells with VN extract strongly induces increased caspase-3 activity as shown in Figure 9.

This high antiproliferative effect of VN was related to the presence of bioactive compounds such as alkaloid, flavonoids luteolin-7-glucoside, casticin, iridoid, glycosides, an essential oil and other constituents like ascorbic acid, carotene, glucononital, benzoic acid, β-sitosterol and glycoside [7]. These results are consistent with previous study which indicated that glycosides and flavones compounds possessing potent anticancer properties against MCF-7 human breast cancer cells [39].

Apoptosis represents an effective way to alleviate damaged cells through the activation of caspase and to balance the cellular proliferation [4]. Human caspase cascade is involved in chemical-induced apoptosis, caspase-3 may cleave essential cellular proteins or activate additional caspases by proteolytic cleavage [36].

To understand the molecular mechanism of VN induced growth inhibition, we found that there was a marked increase in the activation of caspase-3, suggesting that caspase dependent apoptotic death could be another mechanism for the beneficial effects of VN, because it is well established that activation of caspase lead to degradation of cellular proteins, cell shrinkage, DNA fragmentation, loss of plasma membrane potential and membrane blebbing [40]. The activation of caspase-3 induced chromosomal DNA break and finally the occurrence of apoptosis [41,42].

In the present investigation, VN extract showed the activation of caspase-3 enzyme mediated apoptosis in HepG2 cells, and this might due to the presence of glycosides and flavones. This result is in agreement with a previous report which showed that certain products from plants can induce apoptosis in cancerous cells like OCM-1, MCF-7 and HT-29 [39].

## Conclusions

PASS-prediction of VN activity has successfully applied and efficiently helped in selecting the most promising pharmaceutical leads with required properties and high accuracy. It would save unnecessary wastage of chemicals and time by avoiding random plant selection methods. It can be seen from the results of PASS that most probable activities are antioxidant, antiproliferative and hepatoprotectants. Therefore, the present approach can be very useful in plant prediction activity according to their required properties without undesirable side effects. This study strongly suggests that VN extract significantly enhanced antioxidant activity and proposed a tumour preventive action against HepG2 cell lines at a dose and time-dependent manner but with lower toxicity toward WRL68 cells. In addition, the morphological analysis using AO/EB staining procedure revealed that the growth and proliferation inhibition is through proteolytic cleavage of caspase-3 protein and intrinsic apoptosis pathway. However, further studies are needed to determine the molecular mechanisms of the active components and to evaluate the potential *in vivo* anticancer activity of VN extract.

## Competing interests

The authors declare that they have no competing interest.

## Authors’ contributions

Conceived and designed the experiments: FK, NMK, MAA and WAY. Performed the experiments FK, WAY. Analyzed the data: FK and WAY. Contributed reagents, materials and analysis tools: FK, NMK, MAA and WAY. Wrote the paper: FK and WAY. Editing the paper: FK, NMK, MAA and WAY. All authors read and approved the final manuscript.

## Pre-publication history

The pre-publication history for this paper can be accessed here:

http://www.biomedcentral.com/1472-6882/13/343/prepub
